# All-trans retinoic acid arrests cell cycle in leukemic bone marrow stromal cells by increasing intercellular communication through connexin 43-mediated gap junction

**DOI:** 10.1186/s13045-015-0212-7

**Published:** 2015-10-07

**Authors:** Yao Liu, Qin Wen, Xue-lian Chen, Shi-jie Yang, Lei Gao, Li Gao, Cheng Zhang, Jia-li Li, Xi-xi Xiang, Kai Wan, Xing-hua Chen, Xi Zhang, Jiang-fan Zhong

**Affiliations:** Department of Hematology, Xinqiao Hospital, the Third Military Medical University, Xinqiao Street, Chongqing, 400037 China; Department of Pathology, Keck School of Medicine, University of Southern California, Los Angeles, CA 90033 USA

**Keywords:** All-trans retinoic acid, Bone marrow stromal cells, Connexin 43, Gap junctional intercellular communication, Leukemia BMSCs

## Abstract

**Background:**

Gap junctional intercellular communication (GJIC) is typically decreased in malignant tumors. Gap junction is not presented between hematopoietic cells but occurred in bone marrow stromal cells (BMSCs). Connexin 43 (Cx43) is the major gap junction (GJ) protein; our previous study revealed that Cx43 expression and GJIC were decreased in acute leukemic BMSCs. All-trans retinoic acid (ATRA) increases GJIC in a variety of cancer cells and has been used to treat acute promyelocytic leukemia, but the effects of ATRA on leukemic BMSCs is unknown. In this study, we evaluated the potential effects of ATRA on cell cycle, proliferation, and apoptosis of leukemic BMSCs. Effects of ATRA on Cx43 expression and GJIC were also examined.

**Methods:**

Human BMSCs obtained from 25 patients with primary acute leukemia, and 10 normal healthy donors were cultured. Effects of ATRA on cell cycle, cell proliferation, and apoptosis were examined with or without co-treatment with amphotericin-B. Cx43 expression was examined at both the mRNA and protein expression levels. GJIC was examined by using a dye transfer assay and measuring the rate of fluorescence recovery after photobleaching (FRAP).

**Results:**

ATRA arrested the cell cycle progression, inhibited cell growth, and increased apoptosis in leukemic BMSCs. Both Cx43 expression and GJIC function were increased by ATRA treatment. Most of the observed effects mediated by ATRA were abolished by amphotericin-B pretreatment.

**Conclusions:**

ATRA arrests cell cycle progression in leukemic BMSCs, likely due to upregulating Cx43 expression and enhancing GJIC function.

**Electronic supplementary material:**

The online version of this article (doi:10.1186/s13045-015-0212-7) contains supplementary material, which is available to authorized users.

## Background

Gap junctions (GJs) are specialized structures consisting of highly conserved trans-membrane proteins connexins and allow passage of small molecule (<1 kD) between the cytoplasm of two adjacent cells [1, 2]. A total of 21 human connexin subtypes have been identified up to date, with each one displaying unique tissue expression pattern [3]. Decreased expression of connexins and thus compromised gap junctional intercellular communication (GJIC) is noted in a variety of malignant tumors and contributes to their uncontrolled proliferation [1, 3–5]. Increased connexin expression and restored GJIC using molecular approach have been shown to inhibit the growth of solid tumors [6–9], decrease invasive potential, and jeopardize neovascularization [10, 11].

GJs do not exist between hematopoietic cells but are present between bone marrow stromal cells (BMSCs) and hematopoietic cells [12, 13]. Communication between BMSCs and hematopoietic cells is an important part of the microenvironment for establishment and growth of tumor cells [14–16]. For GJs at these sites, connexin 43 (Cx43) is the major subtype [12]. Functional GJIC in BMSCs has direct impact on the proliferation and differentiation of hematopoietic stem/progenitor cells [17]. Our previous studies revealed decreased Cx43 expression and GJIC function of acute leukemic BMSCs, which could recover after adopting effective chemotherapy or transfection with Cx43 gene [18, 19]. These findings suggested that Cx43 expression and GJIC function in BMSCs are likely to be a dynamic process and regulated by many factors in vivo and in vitro. This hypothesis has been verified by some studies which demonstrated that some hormones, cytokines, and drugs could affect the expression of Cxs between coupling cells [20–23].

All-trans retinoic acid (ATRA) is a natural derivative of vitamin A and could regulate various physiological events, including cell cycle, embryonic development, and cellular differentiation [24]. ATRA could inhibit malignant transformation and produce anti-proliferative effect in several types of tumor cells, possibly through enhancing GJIC function in tumor cells [25–27]. ATRA has been used in the treatment of acute promyelocytic leukemia (APL) [28] and other hematologic diseases [29, 30] based on induction of differentiation and inhibition of growth. However, whether ATRA affects GJIC in leukemic BMSCs remains unknown.

In this study, we examined the potential effects of ATRA on cell cycle progression of leukemic BMSCs from 25 patients with acute leukemia. Increased Cx43 expression by ATRA was verified with mRNA and Western blot. Enhanced GJIC function was verified with fluorescent dye dispersion as well as a bleaching assay. The experiments revealed arrested cell cycle progression, inhibited growth, and increased apoptosis upon ATRA exposure. These findings suggest that GJIC function is implicated in the therapeutic action of ATRA for leukemia.

## Results

### ATRA increases Cx43 mRNA

**Fig. 1:**
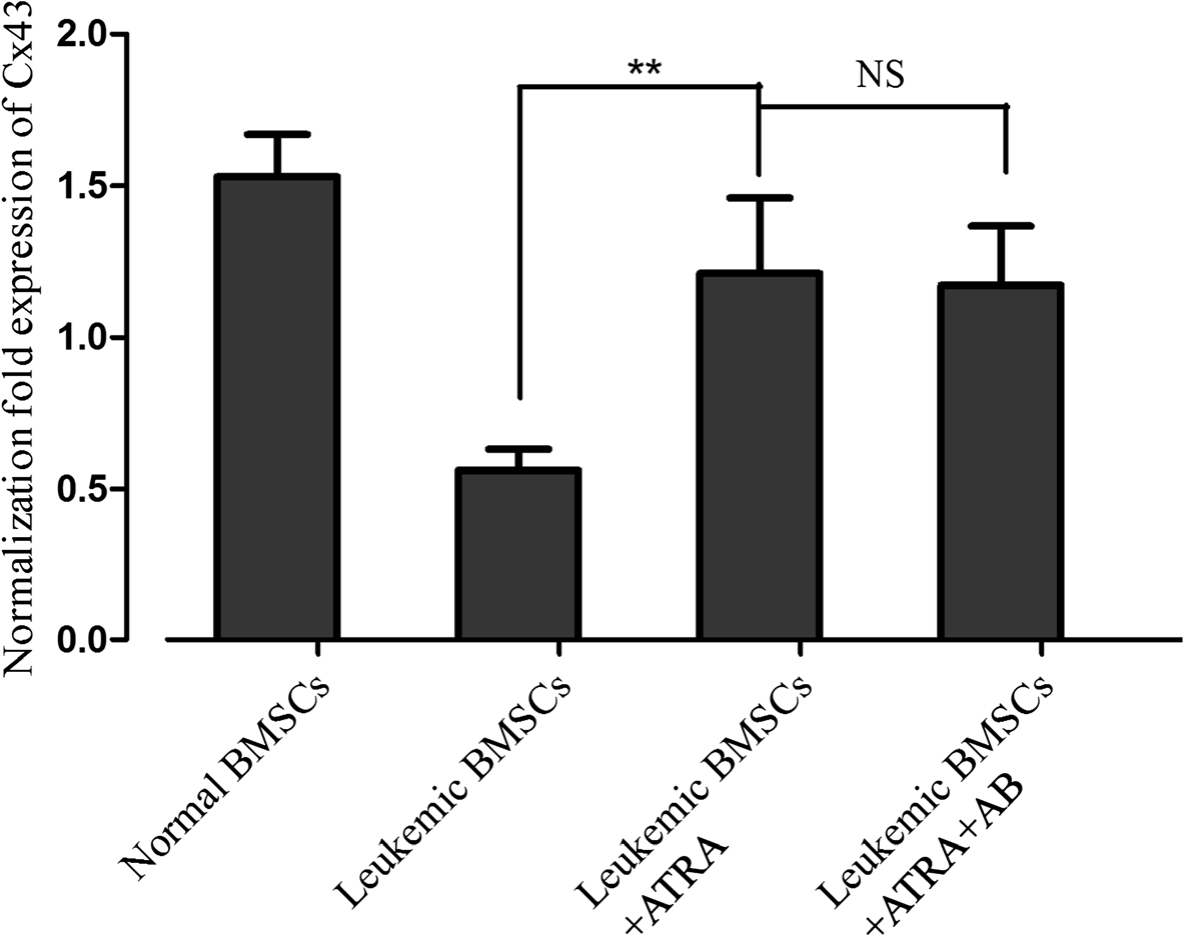
Effects of ATRA and amphotericin-B pretreatment on Cx43 mRNA level in leukemic BMSCs. Cx43 mRNA (normalized against β-actin) was 1.53 ± 0.14 in healthy controls (*N* = 10), 0.56 ± 0.07 leukemic BMSCs (*N* = 10) treated with DMSO, 1.21 ± 0.36 in leukemic BMSCs (*N* = 25) exposed to ATRA (10 uM), and 1.17 ± 0.196 in leukemic BMSCs exposed to ATRA + amphotericin-B. Data are presented as mean ± SD. *Double asterisks* indicate *P* < 0.01

Quantitative reverse transcription polymerase chain reaction (qRT-PCR) analysis showed that treatment with ATRA (10 uM) increased Cx43 mRNA levels in leukemic BMSCs by >50 % (*p* < 0.01) to a level closed to normal BMSCs (*p* < 0.05, Fig. 1). Pretreatment with amphotericin-B did not affect the ATRA action on Cx43 mRNA.

### ATRA increases Cx43 protein expression

**Fig. 2:**
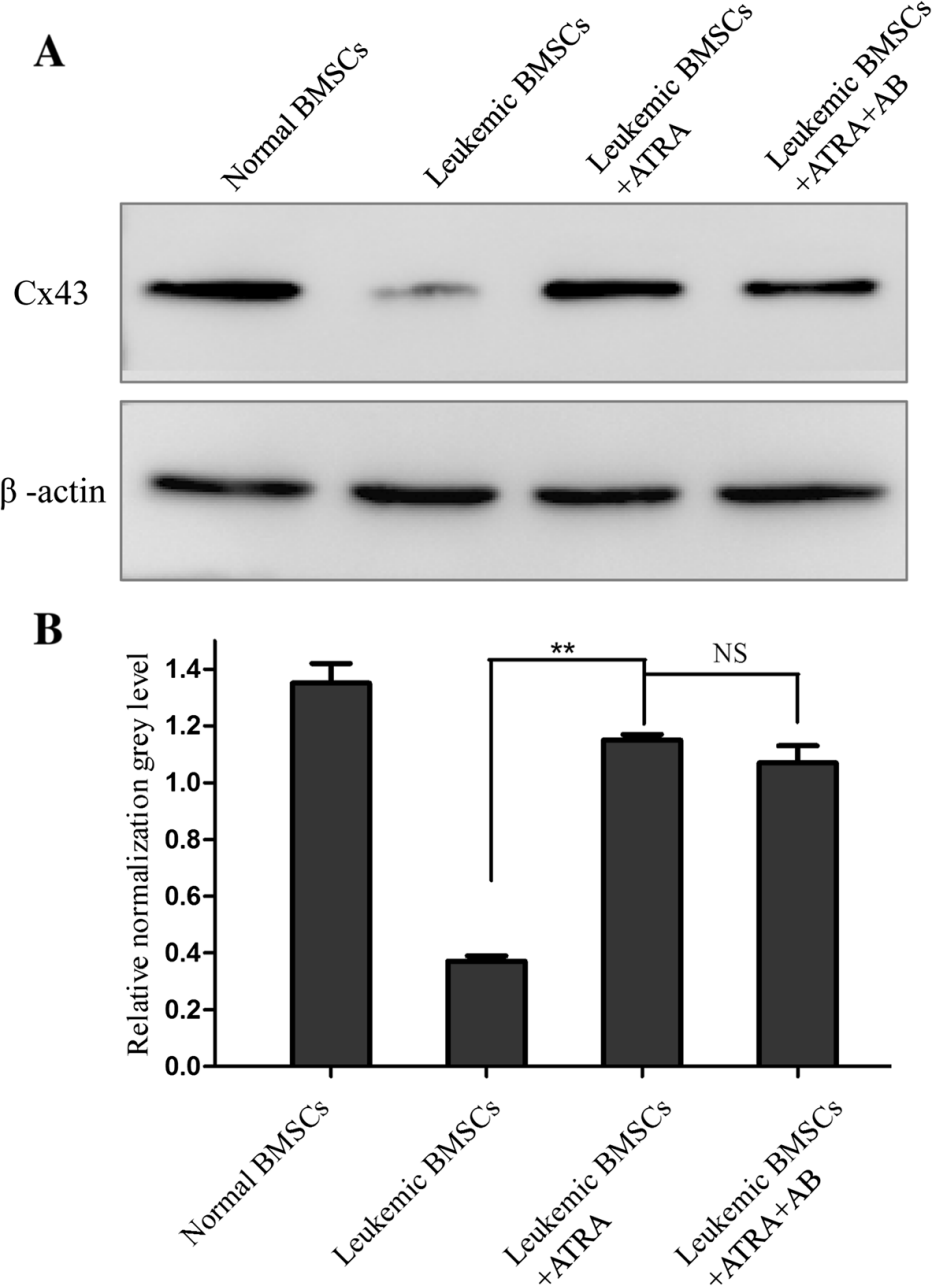
Effects of ATRA and amphotericin-B pretreatment on Cx43 protein level in leukemic BMSCs. **a** Cx43 protein (normalized against β-actin) was 1.35 ± 0.17 in healthy controls (*N* = 10), 0.37 ± 0.02 in leukemic BMSCs (*N* = 25) treated with DMSO, 1.15 ± 0.03 in leukemic BMSCs exposed to ATRA (10 uM) (*N* = 25), and 1.07 ± 0.02 in leukemic BMSCs treated with both ATRA and amphotericin-B (*N* = 25); **b** relative normalization gray level of Cx43 Western blotting band in health controls, leukemic BMSCs, leukemic BMSCs + ATRA, leukemic BMSCs + ATRA + AB. Data are presented as mean ± SD. *Double asterisks* indicate *P* < 0.01

Western blot analysis showed that ATRA (10 uM) increased Cx43 protein levels by approximately 70 % (*p* < 0.01) to a level not statistically different from normal BMSCs (Fig. 2a, b). Moreover, treatment with amphotericin-B did not affect Cx43 protein expression levels mediated by ATRA.

### ATRA enhances GJIC

**Fig. 3:**
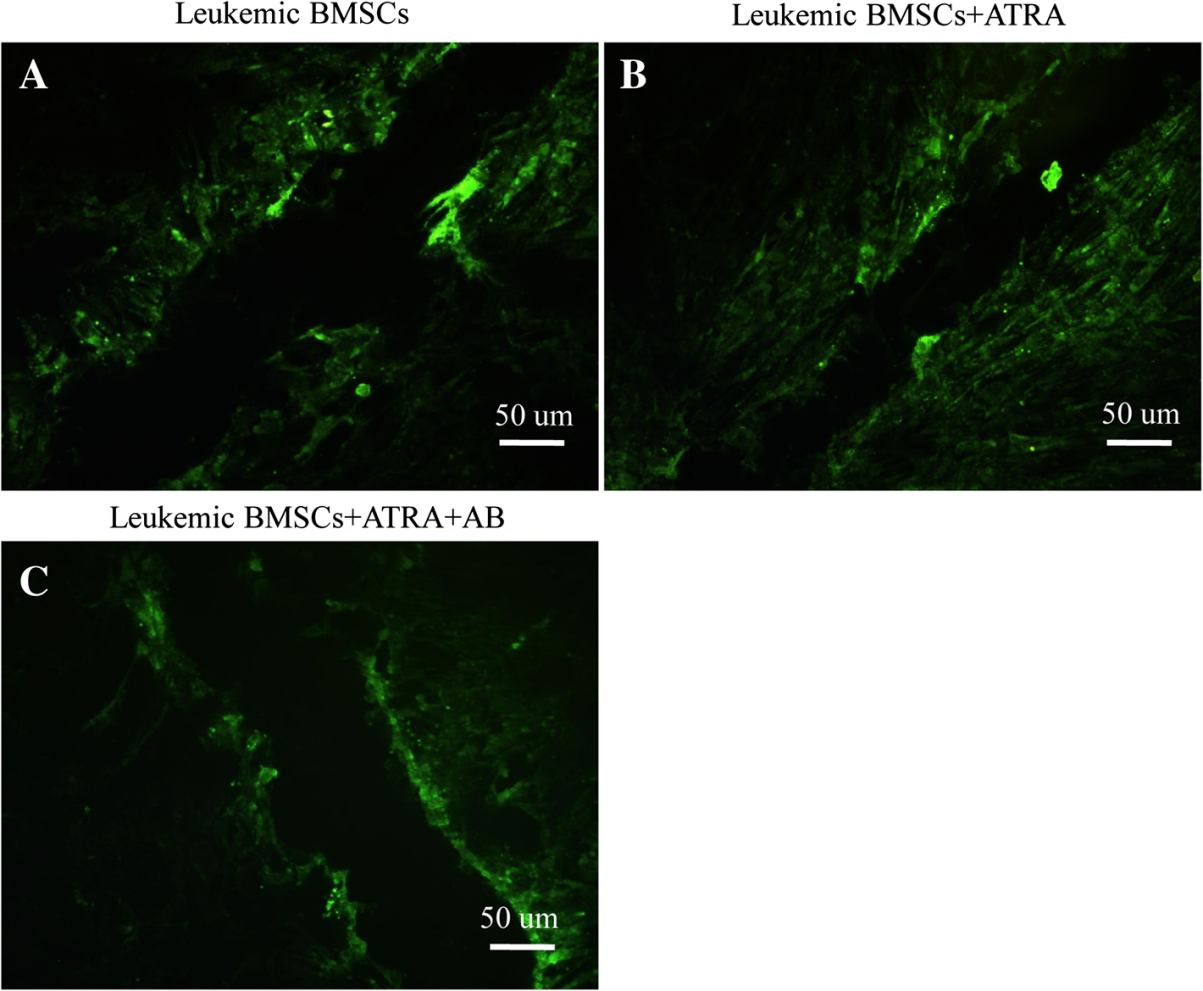
Effects of dye transfer between BMSCs by using laser confocal microscopy. **a** Leukemic BMSCs not exposed to ATRA; **b** leukemic BMSCs exposed to ATRA (10 M); **c** leukemic BMSCs treated with both ATRA and amphotericin-B. Scale bar = 50 um

Dye transfer assay was a rapid, sensitive method to detect GJIC function between cells [31]. In this study, Lucifer yellow (LY) fluorescence in leukemic BMSCs dispersed to only 1–2 cell lines surrounding the wounded edge (Fig. 3a). After ATRA treatment, LY fluorescence dispersed to 4–5 cell lines (Fig. 3b). Pretreatment with AB could block the effect mediated by ATRA (Fig. 3c).

**Fig. 4:**
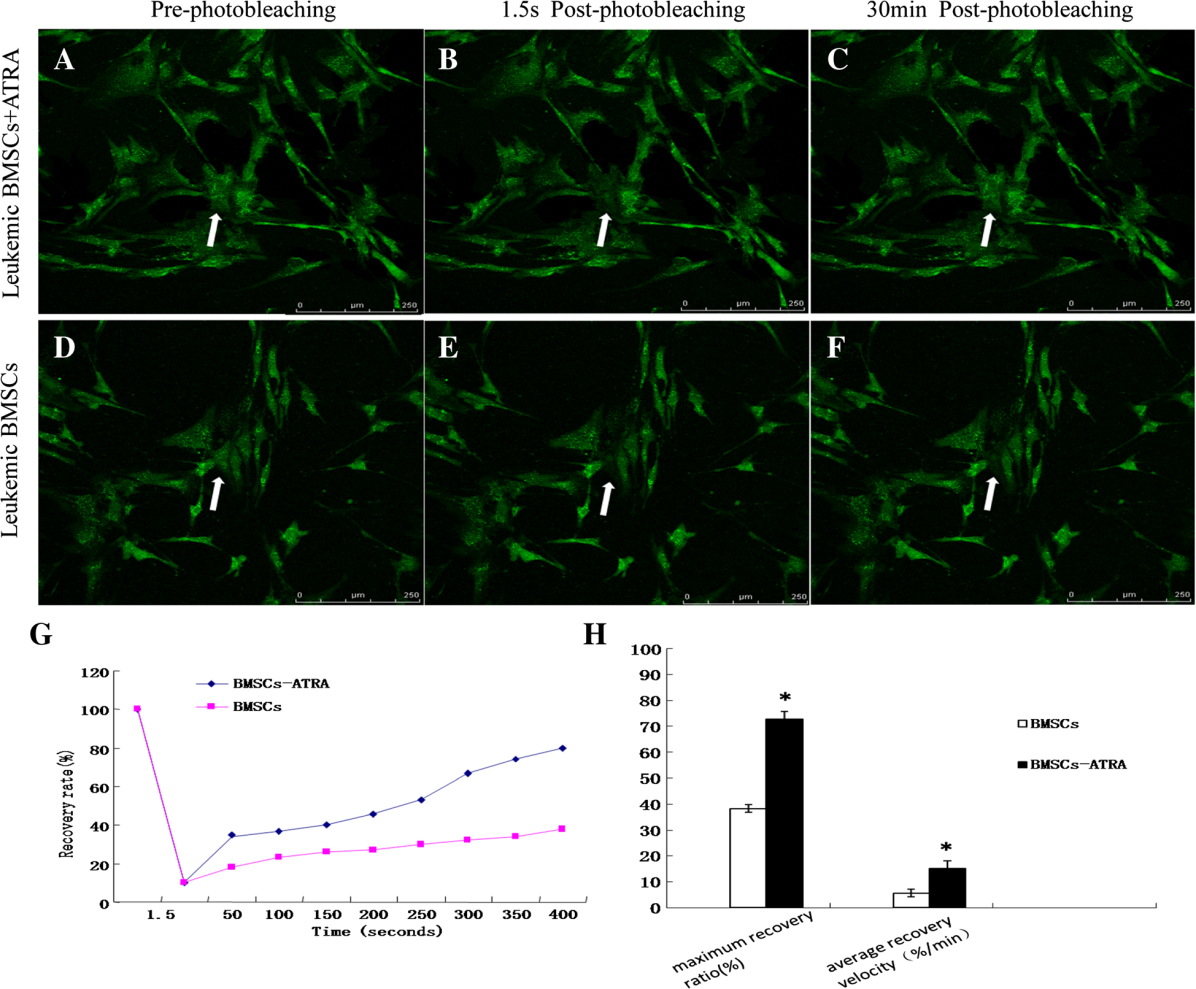
FRAP assay in BMSCs. **a** Pre-photobleaching of leukemic BMSCs exposed to ATRA; **b** 1.5 s post-photobleaching of leukemic BMSCs exposed to ATRA; **c** 30 min post photobleaching of leukemic BMSCs exposed to ATRA. **d**–**f** Leukemic BMSCs without ATRA treatment (**d** pre-photobleaching of leukemic BMSCs without ATRA treatment; **e** 1.5 s post-photobleaching of leukemic BMSCs without ATRA treatment; **f** 30 min post photobleaching of leukemic BMSCs without ATRA treatment). **g** Effects of ATRA on fluorescence redistribution in leukemic BMSCs. **h** Effects of ATRA on GJIC in leukemic BMSCs. *Arrow symbols* photobleached cells. *p < 0.01 vs. leukemic BMSCs. Images were taken under a laser confocal microscope at ×400 magnification

The fluorescence recovery after photobleaching (FRAP) assay showed 20 % recovery of fluorescent signal in leukemic BMSCs at 1 min after photobleaching. The maximal recovery percentage was 36.25 % (±2.14); the average recovery rate was 4.82 % (±0.78) min^−1^ (Fig. 4d–f). ATRA treatment promoted the recovery of fluorescent signal after photobleaching (Fig. 4a–c): 35 % of cells could be recovered in 1 min and 80.94 % (±1.46) could be recovered within 30 min. The average recovery rate was increased to 15.03 % (±1.27) min^−1^ (Fig. 4g, h). Pretreatment with amphotericin-B blocked the effects of ATRA.

### ATRA changes biologic characteristics of BMSCs through GJIC

**Fig. 5:**
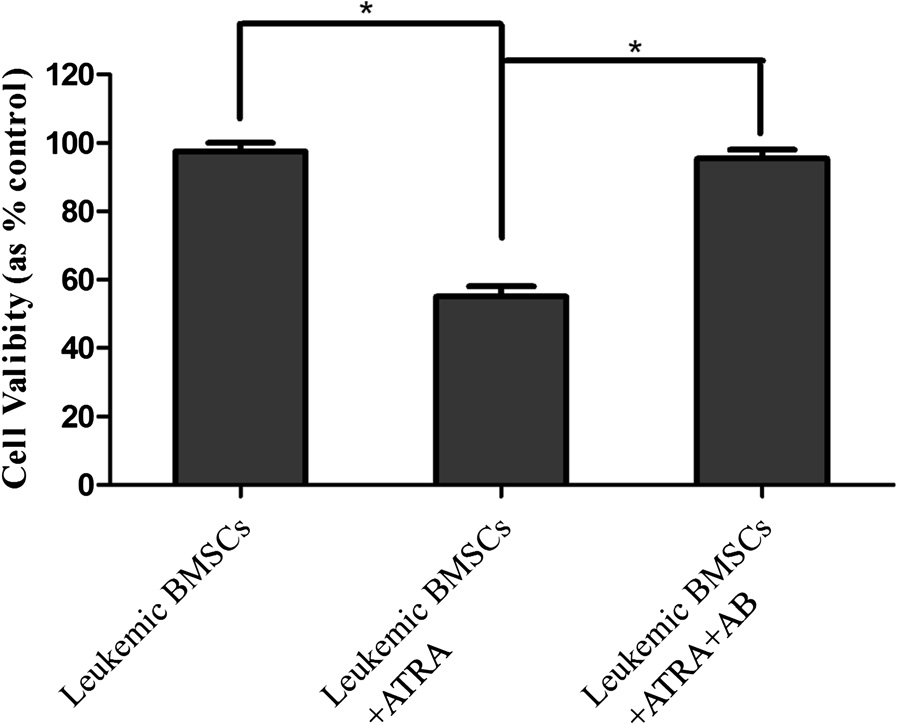
Effects of ATRA on the cell viability of BMSCs (MTT assay). Cell viability was evaluated by MTT assay in leukemic BMSCs, leukemic BMSCs treated with ATRA, and leukemic BMSCs treated with both ATRA and amphotericin-B. Data are presented as mean ± SD. *Single asterisk* indicates significant difference (*P* < 0.05)

ATRA (10 uM for 48 h) inhibited the cell growth of leukemic BMSCs by 40 % (*p* < 0.05; Fig. 5), and pretreatment with amphotericin-B blocked the ATRA effects.

**Fig. 6:**
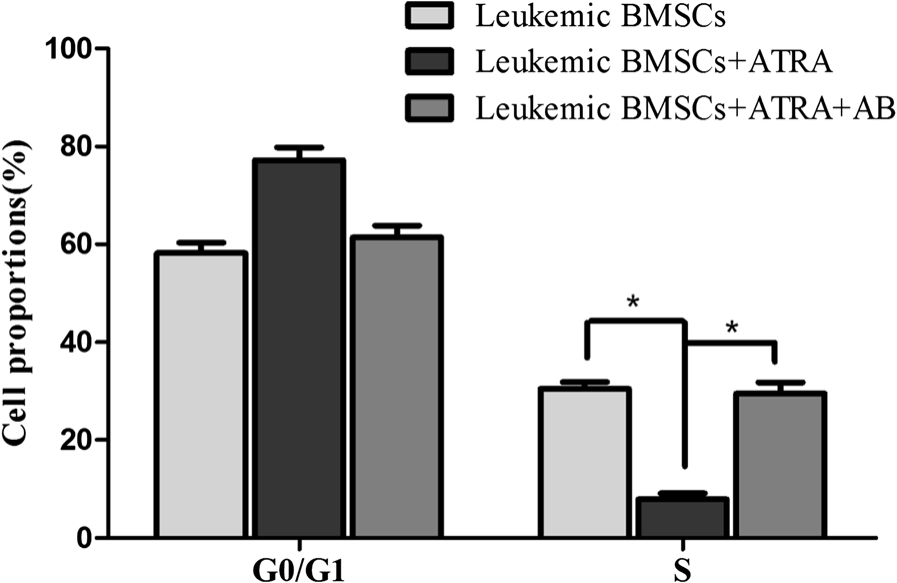
Effects of ATRA on the cell cycle (FCM assay). Cell cycle was examined with FCM assay in leukemic BMSCs, leukemic BMSCs treated with ATRA, and leukemic BMSCs treated with both ATRA and amphotericin-B. Data are presented as mean ± SD. *Single asterisk* indicates significant difference (*P* < 0.05)

Flow cytometric analysis showed that ATRA treatment led to decrease the percentage of the cells in S phase from 29.5 to 7.96 % and led to increase the percentage of cells in the G0/G1 phase from 61.1 to 77.2 % (Additional file 1: Figure S1A–C). Such effects were abolished by amphotericin-B pretreatment (Fig. 6).

**Fig. 7:**
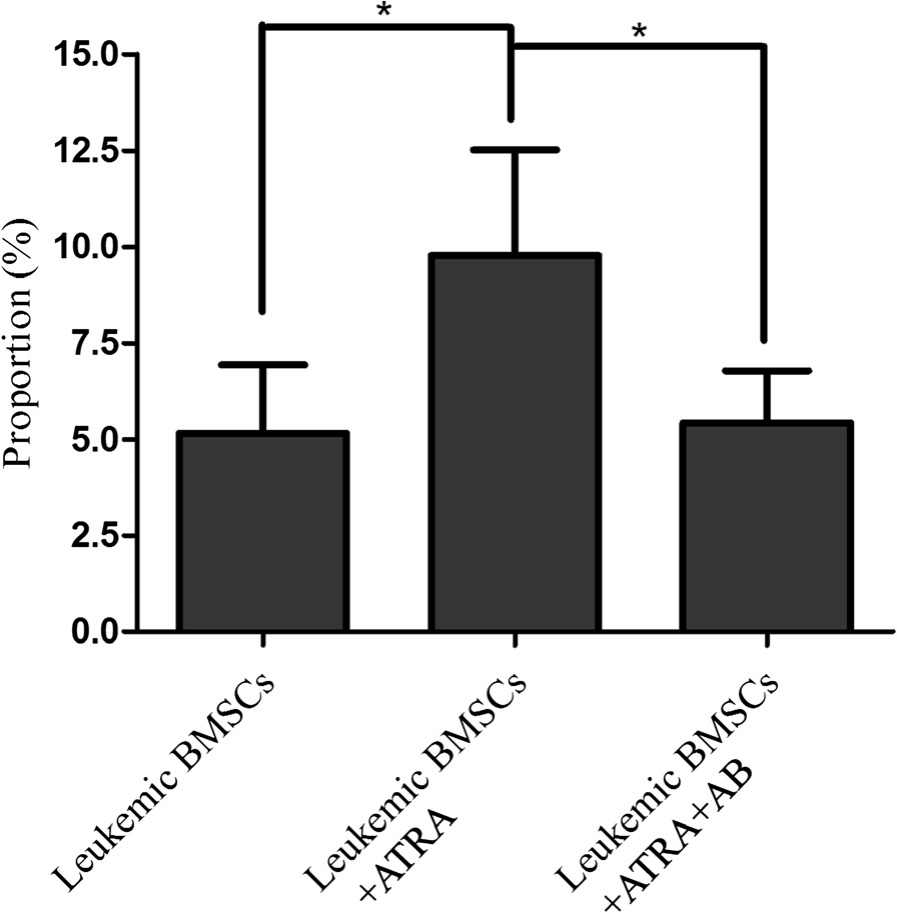
Effects of ATRA on the apoptosis of BMSCs (FCM assay). Cell apoptosis was examined with FCM assay in leukemic BMSCs, leukemic BMSCs treated with ATRA, and leukemic BMSCs treated with both ATRA and amphotericin-B. Data are presented as mean ± SD. *Single asterisk* indicates significant difference (*P* < 0.05)

ATRA treatment increased apoptosis rate from 5.16 ± 2.35 to 9.78 ± 4.88 % (*p* < 0.05) (Additional file 2: Figure S2A–C). Such effects were attenuated by amphotericin-B pretreatment (*p* < 0.05; Fig. 7).

## Discussion

Abnormal expression of connexin genes in tumor cells is the result of downregulation rather than genomic deletion or mutation, and thus could be rescued by pharmacological treatment [32, 33]. Increasing the expression of connexin genes could restore the function of gap junctions [3, 34]. Recent studies showed that ATRA could improve GJIC function in several types of tumor cells [28, 35].

In the current study, we extended previous findings to leukemic BMSCs. ATRA treatment increased the expression of Cx43 at both mRNA and protein levels and resulted in increased intercellular communication. The mechanisms of such action remain obscure, but based on the secondary structure of Cx43 [36, 37], it is reasonable to speculate that ATRA could bind to retinoic acid nuclear receptors (i.e., RARs and RXRs) and activate the PKC pathways, which in turn phosphorylate proteins that bind to the phosphorylation site of the Cx43 mRNA. The resulting instability due to AQUA repeated sequences decrease the degradation of Cx43 mRNA. Alternatively, ATRA could activate Cx43 gene transcription and translation more directly through binding to factors such as AP-1.

In a previous study from this laboratory [18], we demonstrated that GJIC function is dysfunctional between leukemic BMSCs. Such a finding is consistent with the reported decrease in GJIC in several solid tumors [8, 38, 39]. In the current study, we showed that GJIC between leukemic BMSCs is enhanced by ATRA treatment. It is noteworthy to mention the discrepancy of the reported action of ATRA on connexin gene expression in tumors in previous studies. For example, Wageenblatt et al. [40] reported inhibited expression of several connexin genes, including Cx43, by ATRA in carcinoma of urinary bladder. Based on such a finding, the authors argued that connexin gene upregulation provides a “growth advantage” in certain type(s) of tumor cells.

The results of the current study clearly showed that ATRA could arrest cell cycle progression in leukemic BMSCs. Consistently, cell proliferation was decreased and apoptosis was enhanced. These effects could be abolished by amphotericin-B pretreatment, and thus could reasonably attributed to GJICs.

The connection between increased GJIC and BMSC biological properties is now unknown. Potential mechanisms could include purinergic signaling via extracellular adenosine triphosphate (ATP), change of Ca^2+^ internal flow from extracellular through gap junction, and downregulation of monocyte chemotactic protein 1 expression [41–44]. Nevertheless, the current study extended previous findings of ATRA in solid tumors [11, 45] to leukemia.

## Conclusions

ATRA treatment could arrest cell cycle progression in leukemic BMSCs, likely due to increasing Cx43 expression and enhancing GJICs in human leukemic BMSCs. These findings encourage future exploration of using ATRA in the treatment of hematopoietic malignancies.

## Materials

### Sample source

Samples of acute leukemic bone marrow were obtained from 25 patients with primary acute leukemia from the Department of Hematology of Xinqiao Hospital between April 2014 and February 2015. Patients included 14 males and 11 females, with a median age of 33. Among the patients were 14 patients with ALL, 3 patients with AML-M2, 3 patients with AML-M3, 3 patients with AML-M5, and 2 patients with AML-M6. The diagnosis was established on the basis of clinical data, tissue/cell morphology, and immunophenotyping. Bone marrow samples from ten normal healthy donors were used as healthy controls. The research was approved by the Ethics Committee of Xinqiao Hospital, and all subjects gave written informed consent.

### Cell culture and treatment

Human BMSCs were isolated as described previously [46] and were plated in T75 flasks for continuous passage in Dulbecco’s modified Eagle’s medium (DMEM) (Gibco, USA) supplemented with 12.5 % FBS (Gibco, USA), 12.5 % HS (Gibco, USA), and 0.1 mM hydrocortisone (Sigma, USA). Medium was changed twice weekly, and cells were passaged into fresh culture flasks at a ratio of 1:4 upon reaching confluence by using trypsin (Sigma, USA). The cultures were incubated at 37 °C in a humidified incubator with 5 % CO_2_.

ATRA and amphotericin-B (both from Sigma) stock solutions were prepared in dimethyl sulfoxide (DMSO; Sigma, USA) and stored at −20 °C prior to use. The final concentration of DMSO was <0.1 %. BMSCs at passage 3 (reach to 80 % confluence) were used for experiments.

### qRT-PCR

Total RNA was extracted from BMSCs by using a commercial kit from Stratagene (USA). cDNA synthesis and amplification were carried out using 2 μg mRNA with RevertAid™ First Strand cDNA Synthesis Kit (Fermentas, USA) and GeneAmp PCR System 2700 Thermal Cycler (Applied Biosystems, USA). qRT-PCR conditions were as follows: 95 °C for 15 min, then 40 cycles of 94 °C for 30 s, 60 °C for 30 s, and 72 °C for 30 s. Primers were as follows: Cx43 (NM_000165), forward 5′-AACCTGGTTGTGAAAATGTC-3′; reverse 5′-GCAAGTGTAAACAGCACTCA-3′; β-actin (NM_001101), forward 5′-CCTGTGGCATCCACGAAACT-3′; reverse 5′-GAGCAATGATCCTGATCTTC-3′. Results were normalized against β-actin.

### Western blot

Western blot analysis was performed using whole-cell extract. Protein concentration of the samples was determined using a BCA method. Proteins (20 μg per lane) were separated using 12 % SDS-PAGE gel, transferred to nitrocellulose membrane, and blocked with 5 % skim milk in PBS. The membrane was incubated with a rabbit polyclonal anti-Cx43 antibody (Sigma, USA), and then with a secondary antibody linked to horseradish peroxidase prior to ECA visualization (Amersham Biosciences AB, Uppsala, Sweden). Blots were washed, reprobed with an anti-GAPDH antibody (Sigma, USA), and then developed in an identical manner for assessment of GAPDH to ensure even loading.

### Dye transfer assay

Transfer assay was carried out as previously described [47]. Briefly, cultured cells were rinsed thoroughly with Hanks’ balanced salt solution (HBSS) (with calcium and magnesium) containing 1 % BSA. A 27-gauge needle was used to create multiple scratches (minimum distance between scratches 1 cm) in the cell monolayer in the presence of PBS containing 0.5 % Lucifer yellow (Gibco, USA). After exactly 1 min, the culture was rinsed with HBSS and then incubated for 1–10 min in the saved culture medium to allow the loaded dye to transfer to adjoining cells. The cells were then rinsed and fixed with 4 % paraformaldehyde and immediately analyzed under a laser confocal scanning microscope (LCSM; Leica, German) at 485 nm. GJIC was defined as the average distance of dye travel (μm) at six different sites in each sample.

### FRAP assay

FRAP assay was carried out in living cells as described previously [48]. Briefly, cells cultured on glass cover slips were loaded with C-FDA (10 μmol/L, 15 min; Sigma) at 37 °C and washed thoroughly with DMEM medium. A cell with direct contact with 4–5 neighboring cells within a cluster was selected under the LCSM. A pre-bleach image of the whole field was taken using low laser intensity (acousto-optic tunable filter (AOTF) = 10 %, zoom = ×2). Then, laser intensity was increased by ×50 (AOTF = 50 %, zoom = ×20), and the target BMSCs was photobleached for 1 s. Transfer of fluorescent dye from neighboring cells was examined by scanning at 50 s intervals for a total period of 30 min. The rate of fluorescence recovery index was calculated as: *R* = (*I*_t_ − *I*_0_) / (*I* − *I*_0_) × 100 %, where *I*_0_ is the intensity of the photo-bleached fluorescence and *I* is the intensity of pre-bleached fluorescence.

### MTT assay

Cell proliferation was examined using a methyl thiazolyl tetrazolium (MTT) assay (Sigma, USA) at 610 nm. Data were averaged from three independent experiments.

### Flow cytometry

Flow cytometry (FCM) assay was carried out as described previously [49]. BMSCs were washed, fixed in 70 % ethanol, and resuspended in 10 mL PBS. Cells were stained with propidium iodide (5 μL 10 mg/mL) and DNAse-free RNase (200 μg/mL) for 20 min prior to FACS analysis using a FACSVantage flow cytometer (Becton Dickinson, USA) and analyzed by CellQuest software. At least 1 × 10^4^ cells were analyzed for each sample.

### Apoptosis

Apoptosis was determined by Annexin V-FITC (Gibco, USA) and FCM analyses. After washing with PBS, 10^6^ BMSCs were resuspended in binding buffer containing Annexin V-FITC (1 mg/mL). The mixture was incubated for 10 min in the dark under room temperature and then analyzed with FACSVantage flow cytometer and CellQuest software.

### Statistical analysis

Data are represented as mean with standard deviation and analyzed with Student’s *t* test, except for GJIC (Pearson’s chi-squared test). Statistical significance was set at *p* < 0.05.

## Additional files

Additional file 1: Figure S1. The original flow plots of cell cycle in leukemic BMSCs using FCM assay. (A) Leukemic BMSCs; (B) leukemic BMSCs exposed to ATRA; (C) leukemic BMSCs treated with both ATRA and amphotericin-B. Additional file 2: Figure S2. The original flow plots of cell apoptosis in leukemic BMSCs using FCM assay. Leukemic BMSCs were treated DMSO, ATRA, and ATRA + AB, then cells were stained with Annexin V-FITC and propidium iodide (PI), followed by analysis on a flow cytometer. (A) Leukemic BMSCs; (B) leukemic BMSCs exposed to ATRA; (C) leukemic BMSCs treated with both ATRA and amphotericin-B.

## Abbreviations

AOTF: acousto-optic tunable filter
ATP: adenosine triphosphate
ATRA: all-trans retinoic acid
BMSCs: bone marrow stromal cells
Cx43: connexin 43
DMSO: dimethyl sulfoxide
FCM: flow cytometry
FRAP: fluorescence recovery after photobleaching
GJIC: gap junctional intercellular communication
LCSM: laser confocal scanning microscope
LY: Lucifer yellow
MTT: methyl thiazolyl tetrazolium
qRT-PCR: quantitative reverse transcription polymerase chain reaction
