# Supplementary figures and images for: All-trans retinoic acid arrests cell cycle in leukemic bone marrow stromal cells by increasing intercellular communication through connexin 43-mediated gap junction
Source: J Hematol Oncol. 2015 Oct 7;8:110. doi: 10.1186/s13045-015-0212-7 (PMC4597383; doi:10.1186/s13045-015-0212-7)

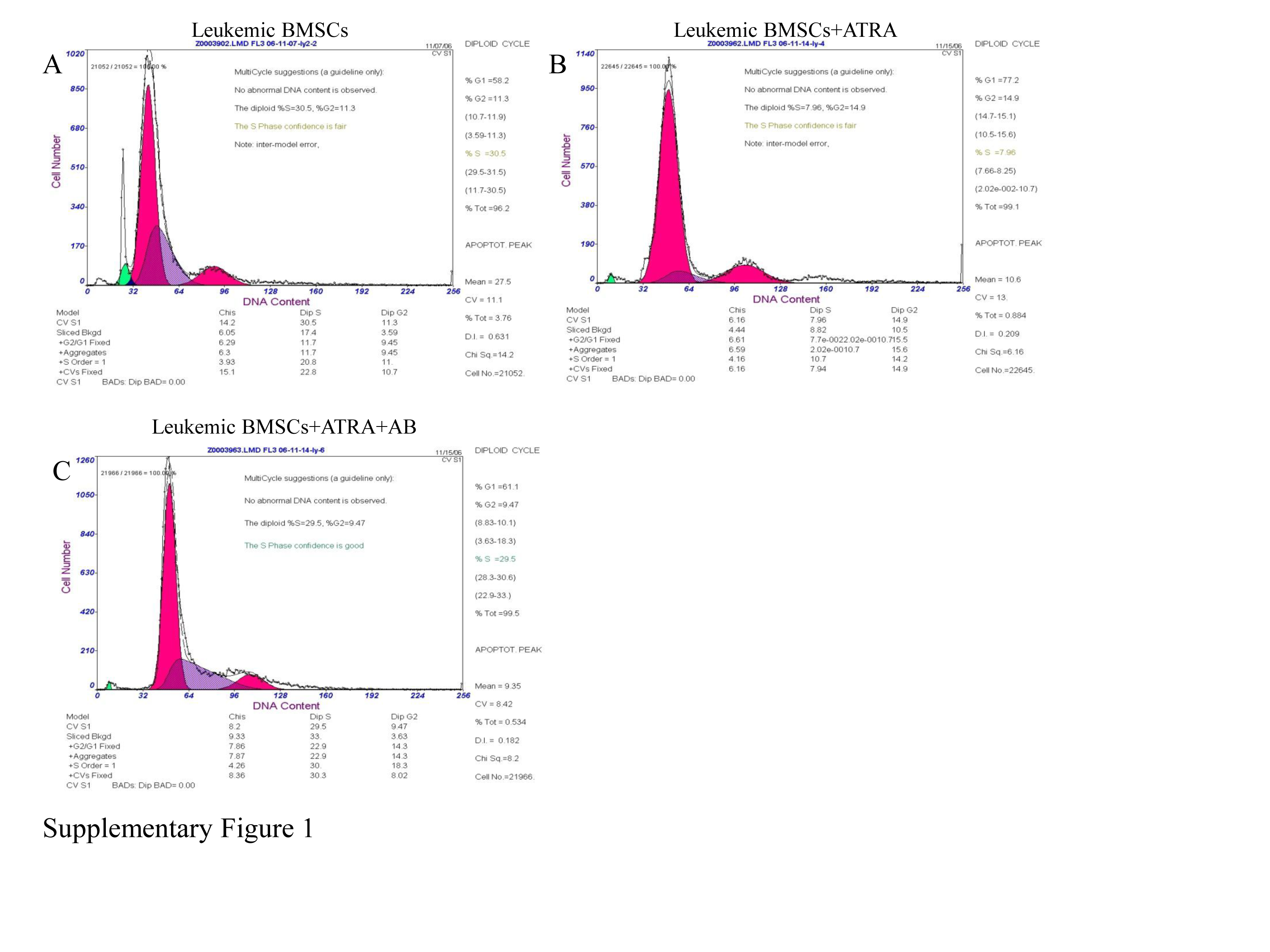

Supplement: Additional file 1: Figure S1. — The original flow plots of cell cycle in leukemic BMSCs using FCM assay. (A) Leukemic BMSCs; (B) leukemic BMSCs exposed to ATRA; (C) leukemic BMSCs treated with both ATRA and amphotericin-B. [file 13045_2015_212_MOESM1_ESM.tif]

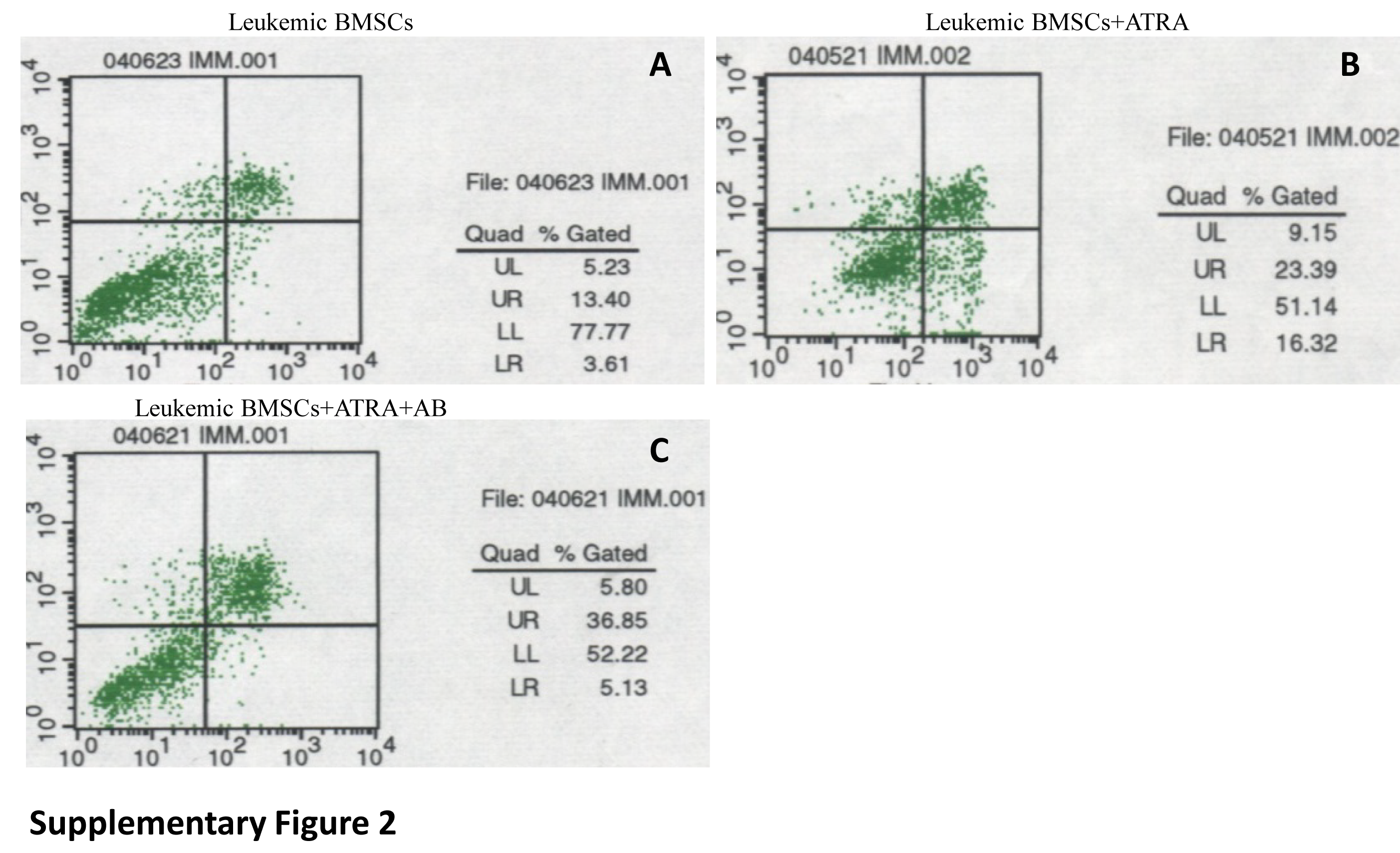

Supplement: Additional file 2: Figure S2. — The original flow plots of cell apoptosis in leukemic BMSCs using FCM assay. Leukemic BMSCs were treated DMSO, ATRA, and ATRA + AB, then cells were stained with Annexin V-FITC and propidium iodide (PI), followed by analysis on a flow cytometer. (A) Leukemic BMSCs; (B) leukemic BMSCs exposed to ATRA; (C) leukemic BMSCs treated with both ATRA and amphotericin-B. [file 13045_2015_212_MOESM2_ESM.tif]
